# An Indirect Comparison of the Efficacy and Safety of Dostarlimab and Doxorubicin for the Treatment of Advanced and Recurrent Endometrial Cancer

**DOI:** 10.1093/oncolo/oyac188

**Published:** 2022-09-16

**Authors:** Cara Mathews, Domenica Lorusso, Robert L Coleman, Susan Boklage, Jamie Garside

**Affiliations:** Brown University, Providence, Providence, RI, USA; Fondazione Policlinico Gemelli of Rome, Rome, Italy; Department of Gynecologic Oncology, Catholic University of Sacred Heart, Rome, Italy; Texas Oncology, US Oncology Research, TX, USA; GSK, Collegeville, PA, USA; GSK, London, UK

**Keywords:** endometrial neoplasms, uterine neoplasms, doxorubicin, immune checkpoint inhibitors, DNA mismatch repair, microsatellite instability

## Abstract

**Background:**

There is no clear standard of care for advanced/recurrent endometrial cancer (EC) following platinum-based therapy. Dostarlimab is approved for patients with mismatch repair-deficient (dMMR)/microsatellite instability-high (MSI-H) advanced/recurrent EC. This indirect treatment comparison (ITC) assessed dostarlimab efficacy and safety from the single-arm GARNET (NCT02715284) trial compared with doxorubicin from ZoptEC (NCT01767155).

**Patients and Methods:**

Patient-level data and study variables from GARNET Cohort A1 (dMMR/MSI-H EC) and the ZoptEC doxorubicin control arm were merged. Patients were matched based on eligibility criteria (main analysis population). Safety population included all patients who received treatment. The primary efficacy comparison outcome, overall survival (OS), was calculated using a Cox proportional hazards model, with adjusted stabilized inverse probability of treatment weighting. Modified assessment-scheduled matching Kaplan--Meier analysis was used for progression-free survival (PFS) and time to deterioration (TTD) in quality of life (QoL).

**Results:**

In the main analysis population, median (95% CI) OS was not reached (NR; 18.0 months--NR) for dostarlimab (*n* = 92) and was 11.2 (10.0-13.1) months for doxorubicin (*n* = 233; HR: 0.41 [95% CI: 0.28-0.61]); median PFS was 12.2 (3.3-NR) and 4.9 (4.1-6.6) months, respectively. Median TTD in QoL was NR (2.5-NR; *n* = 61) and 4.5 (4.1-5.4; *n* = 188) months, respectively. Similar rates of adverse events (AEs, 11.6% vs 15.3%) and serious AEs (34.1% vs 30.1%) were observed with dostarlimab (*n* = 129) and doxorubicin (*n* = 249). Grade ≥3 AEs occurred in 48.1% vs 78.3%, respectively.

**Conclusion:**

This ITC suggests a favorable benefit:risk profile for dostarlimab in patients with dMMR/MSI-H advanced/recurrent EC.

## Teaser

This indirect treatment comparison assessed dostarlimab efficacy and safety compared with doxorubicin in advanced or recurrent endometrial cancer.

**Implications for Practice**

There is no clear guidance for the treatment of advanced/recurrent endometrial cancer after progression on platinum-containing regimens. In the absence of head-to-head comparison of the anti-programmed cell death receptor-1 monoclonal antibody dostarlimab with other treatments in mismatch repair-deficient (dMMR)/microsatellite instability-high (MSI-H) disease, an indirect comparison was performed with the commonly used second-line chemotherapy agent doxorubicin. Dostarlimab demonstrated longer progression-free survival (12.2 vs.4.9 months), 59% lower risk of death, and slower deterioration in quality-of life, with similar rates of adverse events versus doxorubicin, supporting a favorable benefit-risk profile for dostarlimab in patients with dMMR/MSI-H advanced/recurrent endometrial cancer.

## Introduction

Endometrial cancer (EC) is the most common gynecologic malignancy in the United States (US) and European Union (EU), accounting for 5% of all cancers in women,^1-3^ and the incidence is increasing.^4^ Platinum-based doublet chemotherapy is considered the standard of care first-line therapy for patients with newly diagnosed advanced EC or recurrent EC (advanced/recurrent EC), with carboplatin plus paclitaxel being the preferred regimen.^5,6^ For patients with advanced/recurrent EC, there is no clear guidance for second-line treatment.^5,6^ Chemotherapies including carboplatin, paclitaxel, cisplatin, and doxorubicin (including liposomal doxorubicin) have been among the agents commonly used.^7,8^ However, response rates with chemotherapy in these patients are low (10%-15%) and prognosis is poor.^1,9,10^ Additionally, commonly used chemotherapies can be associated with significant toxicity.^11^ Hence, there is an unmet need for novel therapies that are efficacious and tolerable and which maintain, or reduce deterioration in, quality of life.^4^

DNA mismatch repair (MMR) is a process that plays a key role in maintaining genomic stability. Approximately 25-30% of patients with EC have MMR deficiency (dMMR) or high microsatellite instability (MSI-H), which leads to increased mutation rates.^12-14^ It has been hypothesized that there is increased neoantigen production in tumors harboring these mutations, which makes these tumors targets for activated immune cells.^15^ Immune checkpoint inhibitors, such as anti-programmed cell death receptor-1 (PD-1) or anti-programmed death-ligand 1 (PD-L1) therapies, may increase the immune system response to neoantigens produced by these tumors and therefore have the potential to be effective treatments.^16^ In the single-arm, Phase I GARNET trial (NCT02715284), monotherapy with the anti-PD-1 monoclonal antibody (mAb) dostarlimab demonstrated promising efficacy, with an objective response rate (ORR) of 42.3% after a median of 11.2 months follow-up in patients with dMMR/MSI-H advanced/recurrent EC.^17^ Dostarlimab had a manageable safety profile that was consistent with other anti-PD-1 mAbs.^17^ Based on these results, dostarlimab received accelerated approval for the treatment of dMMR/MSI-H advanced/recurrent EC following progression on platinum-based chemotherapy in the US and EU.^18,19^

The efficacy and safety of dostarlimab compared to other commonly used therapies in advanced/recurrent EC has not been evaluated in clinical trials.^17^ In the absence of data from head-to-head trials, data from separate studies with similar designs, definitions, and patient populations can be evaluated using indirect treatment comparisons (ITCs).^20^ Results of ITCs can be combined with clinical efficacy and safety data to inform the value of novel treatments and support health technology assessment submissions.^20^ For example, population-adjusted ITCs are recommended by the National Institute for Health and Care Excellence (NICE) Decision Support Unit.^20^ Multiple single-agent therapies have been evaluated for the treatment of advanced/recurrent EC, including doxorubicin, which has demonstrated clinical activity in patients previously treated with platinum-based chemotherapy.^1,21^ The efficacy of doxorubicin and other chemotherapies does not appear to be influenced by MMR status, as similar clinical outcomes were achieved with treatment of physician’s choice (doxorubicin or paclitaxel) in the MMR proficient (MMRp) cohort and the all-comers cohort (85% MMRp, 15% dMMR) in the KEYNOTE-775 trial of patients with advanced EC with prior platinum exposure.^22^

In the present study, a systematic literature review (SLR) was performed to search for comparator trials for GARNET. Based on general similarities in patient populations, including progression on prior platinum doublet therapy, and study endpoints, the ZoptEC Phase III randomized controlled trial (NCT01767155) of AEZS-108 (zoptarelin doxorubicin) or doxorubicin alone was identified as a relevant clinical trial with a similar patient population to GARNET.^23^ Therefore, an ITC was conducted to inform the comparative efficacy and safety of dostarlimab from GARNET with the doxorubicin control arm from ZoptEC for the treatment of advanced/recurrent EC.

## Patients and Methods

### Inclusion Criteria, Trial Selection, and Data Sources

GARNET is an ongoing multicenter, single-arm, open-label, first-in-human, Phase I clinical trial of dostarlimab in adult patients with advanced solid tumors. GARNET was conducted in 2 parts, consisting of dose finding and safety expansion in Parts 1 and 2A, followed by dose expansion in 5 disease-specific cohorts in part 2B. Cohort A1 included patients with dMMR/MSI-H EC who had an Eastern Cooperative Oncology Group (ECOG) performance status (PS) of ≤1, had progressed on or after platinum-based doublet chemotherapy, and had received no more than 2 lines of chemotherapy for advanced/recurrent disease (key clinical criteria in Supplementary Table S1). Patients received dostarlimab 500 mg intravenously every 3 weeks for the first 4 doses, then 1000 mg every 6 weeks. Further details of the study design, including patient inclusion and exclusion criteria, have been published previously.^17^

A systematic literature review was conducted to search for suitable comparator clinical or observational studies that reported efficacy or safety data on patients with advanced/recurrent EC who were previously treated with platinum-based chemotherapy and were anti-PD-1 naïve.^23^ Based on clinical expert opinion and treatment landscape investigations, 5 primary comparators were identified: paclitaxel, paclitaxel in combination with carboplatin, pegylated liposomal doxorubicin (PLD)/doxorubicin, carboplatin, and cisplatin. The ZoptEC study was a close match to GARNET in terms of patient inclusion criteria and definitions of outcomes.

ZoptEC was a Phase III, multicenter open-label, randomized, active-controlled study which included 511 adult patients with EC who progressed after first-line treatment with a platinum and taxane combination and who had an ECOG PS of ≤2 (key clinical criteria in Supplementary Table S1).^24,25^ In ZoptEC, patients were centrally randomized (1:1) to receive either AEZS-108 (Arm A: 267 mg/m^2^) or doxorubicin (Arm B: 60 mg/m^2^) intravenously every 3 weeks.^24,25^ Unlike in GARNET, MMR/MSI status was not collected for patients in the ZoptEC trial; however, there is not enough evidence to suggest that MMR/MSI status is prognostic or predictive when using chemotherapy in advanced/recurrent EC.^22,26^

Anonymized individual patient data (IPD) were sourced directly from the study sponsors: ZoptEC doxorubicin-arm data from Aeterna Zentaris and GARNET dostarlimab data from GSK.

### Study Populations

The per-protocol safety analysis data set included patients from GARNET Cohort A1 and Arm B from ZoptEC. To make the patient baseline characteristics between the 2 studies comparable, the efficacy analysis data set excluded patients from GARNET if they had previously received >1 prior platinum-based therapy, and from ZoptEC if they had follow-up greater than 36 months or no reported ECOG PS score of 0 or 1. Patient-level data for relevant variables (endpoint, treatment group, and prognostic/treatment-effect modifying variables) from this data set were used to calculate overall survival (OS), progression-free survival (PFS), and ORR. The duration of response (DoR) analysis set included patients who achieved a clinical response. The time to deterioration (TTD) in quality of life (QoL) analysis set included patients with baseline and subsequent QoL data.

### Outcome Measures

The primary endpoint for comparison in this ITC was OS, defined as the time from the first dose of study treatment to death by any cause. In GARNET, patients who did not experience an OS event were censored at their last assessment, and in ZoptEC patients last known to be alive were censored at the date of the last known contact. Secondary endpoints included PFS, ORR, and DoR per Response Evaluation Criteria in Solid Tumors (RECIST) v1.1 (by blinded independent central review in GARNET), adverse events (AEs), and TTD in QoL. PFS was defined as time from the first dose of study treatment to progression or death by any cause. DoR was defined as time from first documentation of overall response (confirmed complete response or partial response) until the time of first documentation of overall response or disease progression or death. TTD in QoL was defined as the time from the first dose of study treatment to the earliest time with a ≥10-point decrease (deterioration) from baseline in global QoL score using the European Organization for Research and Treatment of Cancer Quality of Life Questionnaire (EORTC QLQ-C30). Safety, including serious AEs (SAEs), treatment-emergent AEs (TEAEs) leading to treatment discontinuation, and Grade ≥3 TEAEs, was assessed using the Common Terminology Criteria for Adverse Events (CTCAE) v4.03.

### Inverse Probability of Treatment Weighting (IPTW) Methodology

To control for confounding biases introduced by lack of randomization within this study, treatment effectiveness was calculated for the main analysis data set using IPTW. IPTW is a multi-step estimation procedure involving, firstly, calculating weights for patient characteristics based on how over- or under-represented they are vs, the target population in which these characteristics would be balanced across treatment groups. The weights, based on estimated propensity score, are then used to create a population in which baseline characteristics are independent of treatment assignment; each patient’s weight is equal to the inverse of the probability of receiving the treatment they were assigned. For IPTW calculations, a stabilized weight was used in addition to the standard weight to minimize type I error inflation.

### Assessment-Scheduled Matching

The timings of PFS and DoR assessments varied between GARNET (conducted at weeks 12, 18, 42, 48, and 84) and ZoptEC (conducted at weeks 9, 18, 27, and 32). To adjust for this, a modified assessment-scheduled matching (mASM) approach was utilized, which involved applying an algorithm that used proportional weighting to allocate patients according to distance between nearest assessment visits, and patients were censored at the last doxorubicin visit date +5 weeks.^27^ Sensitivity analyses were performed using non-modified ASM (results not shown). For TTD in QoL, as the patient-reported outcomes were assessed more frequently in GARNET than in ZoptEC, the assessments in this manner were all shifted forward without proportional weighting.

### Statistical Analyses

A Cox proportional hazards regression model with both a standard (non-stabilized) and stabilized IPTW was used to estimate the hazard ratio (HR) for OS of dostarlimab compared with doxorubicin.^28^ The same model was used to estimate the HR for OS for the same population but excluding patients with serous carcinoma (serous-free analysis). For the stabilized IPTW, a proportionality test was conducted to ensure that the proportional HR assumptions were not violated.^29^ Were the assumptions violated, an accelerated time failure model with a Weibull distribution of survival time would be utilized. OS was assessed using Kaplan-Meier analysis between the 2 treatments to determine whether matched comparison should be performed with the stabilized IPTW model; an unadjusted HR of less than 0.65 was required to reach statistical power 0.8 (type I error rate = 0.05 and death rate = 0.6) and justified the use of a matched adjusted comparison. This unadjusted HR was specific to OS since it was the primary outcome of this analysis and determined the assessment feasibility. To identify treatment effect modifiers, the following covariates were used for estimating stabilized IPTW on OS analysis: treatment, candidate for effect modification (age, race, baseline ECOG PS, tumor histology, the International Federation of Gynecology and Obstetrics [FIGO] state, and prior surgery), and the interaction term (treatment x candidate of effect modification). A sensitivity analysis was conducted for OS using the safety analysis data set with stabilized IPTW.

PFS, DoR, and TTD in QoL outcomes were assessed comparatively using Kaplan-Meier analysis and a Cox proportional hazards regression model with IPTW by mASM. Descriptive statistics were used for binary analyses including ORR and safety outcomes; the Clopper-Pearson method was used to calculate 95% confidence intervals (CI) for these analyses. For comparison, the odds ratio (OR) and relative risk (RR) of ORR were assessed using logistic regression with stabilized IPTW. Statistical significance was defined at *P < .*05.

## Results

**Figure 1.**
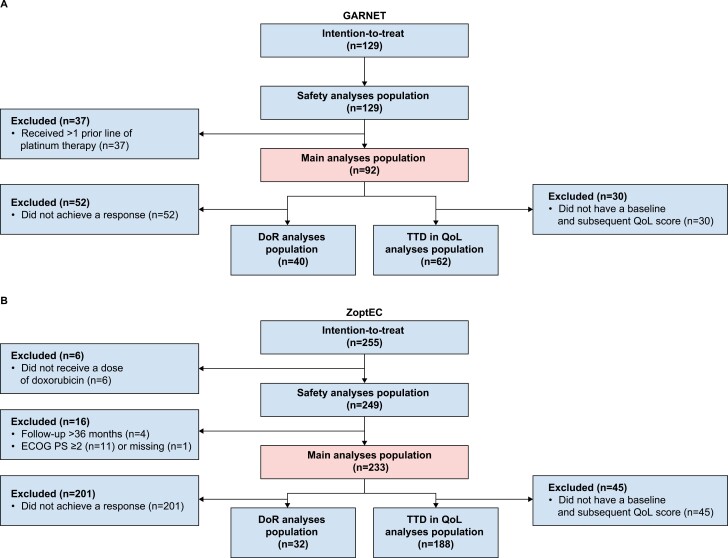
Flow diagram showing patient attrition for each analysis set for (**A**) GARNET and (**B**) ZoptEC.

Abbreviations: DoR, duration of response; ECOG PS, Eastern Cooperative Oncology Group performance status; QoL, quality of life; TTD, time to deterioration.

All patients were included in the safety analysis, which included 129 patients from GARNET and 249 patients from ZoptEC (Fig. 1). The main efficacy analysis data set excluded 37 patients from GARNET, as they had received ≥1 prior platinum-based therapy, and 16 patients from ZoptEC (follow-up greater than 36 months, *n* = 4; ECOG PS score of 2, *n* = 11; missing ECOG score, *n* = 1). Therefore, the main analysis data set consisting of 92 patients from GARNET and 233 matched patients from ZoptEC, and the baseline characteristics for this data set, are shown in Table 1. The mean age of patients in the main analyses set for GARNET and ZoptEC was 63.3 years (standard deviation [SD]: 8.6) and 63.7 years (SD: 8.9), respectively; the most common prior treatment was chemotherapy for GARNET (100%) followed by surgery (90.2%) and was surgery (89.7%) for ZoptEC followed by radiotherapy (54.1%). The most common histology was endometrioid carcinoma type 1 in both populations (68.5% in GARNET and 63.1% for ZoptEC); however, ZoptEC had a higher proportion of patients with serous carcinoma (26.2%) when compared with GARNET (4.3%).

**Table 1.** Baseline characteristics for the main analysis data set.

Characteristic	Dostarlimab (*N* = 92)	Doxorubicin (*N* = 233)
Mean age (SD)	63.3 (8.6)	63.7 (8.9)
Age, n (%)		
<65 years	47 (51.1)	124 (53.2)
≥65 years	45 (48.9)	109 (46.8)
Mean BMI (SD)	29.0 (7.7)^a^	30.8 (8.1)^b^
Prior lines of therapy, *n* (%)^c^		
1	65 (70.7)	NR
2	21 (22.8)	NR
3	4 (4.3)	NR
≥4	2 (2.2)	NR
Prior treatment with, *n* (%)		
Chemotherapy	92 (100)	87 (37.3)
Surgery	83 (90.2)	209 (89.7)
Radiotherapy	65 (70.7)	126 (54.1)
Histology, *n* (%)		
Endometrioid carcinoma type 1	63 (68.5)	147 (63.1)
Serous carcinoma	4 (4.3)	61 (26.2)
Clear cell carcinoma	1 (1.1)	4 (1.7)
Undifferentiated carcinoma	4 (4.3)	0
Mixed carcinoma	1 (1.1)	0
Squamous carcinoma	1 (1.1)	0
Unknown/unspecified	18 (19.6)	21 (9.0)
Disease stage, *n* (%)^d^		
FIGO stage		
I	39 (42.4)	NR
II	7 (7.6)	NR
III	30 (32.6)	NR
IV	16 (17.4)	NR
Advanced (FIGO stage III or IV)	NR	87 (37.3)
Metastatic disease	NR	82 (35.2)
Recurrent disease	NR	64 (27.5)
ECOG PS, n (%)		
0	38 (41.3)	119 (51.1)
1	54 (58.7)	114 (48.9)
Ethnicity, n (%)		
Non-Hispanic or Latino	74 (80.4)	217 (93.1)
Hispanic or Latino	4 (4.3)	13 (5.6)
Unknown	1 (1.1)	3 (1.3)
Not reported	13 (14.1)	0

*n* = 3 patients with missing BMI.

*n* = 4 patients with missing BMI.

The breakdown of treatment history was not available for ZoptEC; however, it can be assumed that 100% of patients had received 1 prior line of therapy, per the study inclusion criteria.

FIGO baseline scores are not directly available within the ZoptEC trial. For IPTW analysis, FIGO Stages III and IV are assumed to equal “Advanced (FIGO Stage III or IV),” as reported in ZoptEC. FIGO Stages I and II are equal to N − Stages III and IV.

Abbreviations: BMI, body mass index; ECOG PS, Eastern Cooperative Oncology Group performance status; FIGO, International Federation of Gynecology and Obstetrics; IPTW, inverse probability of treatment weighting; NR, not reported; SD, standard deviation.

The DoR analysis data set included 40 patients from GARNET and 32 patients from ZoptEC, and the TTD in QoL analysis data set included 62 and 188 patients, respectively.

### Efficacy

**Figure 2.**
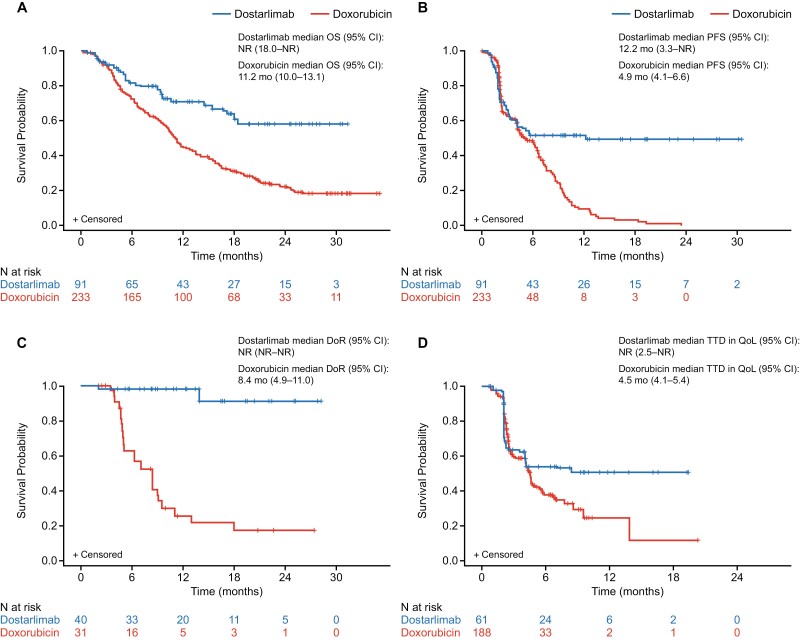
Adjusted* overall survival (**A**), progression-free survival (**B**), duration of response (**C**), and time-to-deterioration in QoL (**D**) for the main analysis data set. Abbreviations: CI, confidence interval; DoR, duration of response; IPTW, inverse probability of treatment weighting; NR, not reached; OS, overall survival; PFS, progression-free survival; QoL, quality of life; TTD, time to deterioration. *The number of patients at risk with IPTW adjustment differs slightly from the total sample size due to weighting by IPTW. The total numbers of patients and those after applying the stabilized IPTW were as follows (dostarlimab/doxorubicin): for OS and PFS, 92/233 and 91/233; for DoR, 40/31 and 40/32; and for TTD in QoL, 62/188 and 61/188. The weighted IPTW number at risk has been rounded to the nearest integer value. As the results of the proportionality test showed a *P*-value greater than .05 (*P = .*096), an adjusted analysis utilizing a Cox proportional hazards model with stabilized IPTW was used for OS analysis.

Dostarlimab was associated with a significantly increased OS compared with doxorubicin, with a 59% lower risk of death (HR: 0.41; 95% CI: 0.28, 0.61; *P < .*0001). A similar HR of 0.46 (95% CI: 0.31, 0.89; *P = .*0001) was seen when the same analysis was performed with the exclusion of all patients with serous carcinoma from both GARNET and ZoptEC (Supplementary Table S2). The median OS with adjusting stabilized IPTW was not reached (NR) with dostarlimab (95% CI: 18.0, NR) and was 11.2 months (95% CI: 10.0, 13.1) for doxorubicin (Fig. 2A). Sensitivity analysis using the safety analysis set provided a similar HR (0.40; 95% CI: 0.28, 0.58) to the main analysis set. There was no evidence of impact of effect modifiers on the OS analysis. PFS was also significantly longer for dostarlimab than doxorubicin, with adjusted (using mASM and stabilized IPTW) median PFS of 12.2 (95% CI: 3.3, NR) and 4.9 (95% CI: 4.1, 6.6) months, respectively (HR: 0.38; 95% CI: 0.28, 0.51; *P < .*0001; Fig. 2B).

Patients who received dostarlimab had a better clinical response than those treated with doxorubicin, with ORRs of 43.5% (95% CI: 33.2, 54.2) and 13.7% (95% CI: 9.3, 18.3), respectively (adjusted OR: 0.19 [95% CI: 0.11, 0.33; *P < .*0001], and RR: 0.30 [95% CI: 0.20, 0.44; *P < .*0001], doxorubicin vs dostarlimab). Median DoR was NR (95% CI: NR, NR) for dostarlimab and 8.4 months (95% CI: 4.9, 11.0) for doxorubicin (HR: 0.04; 95% CI: 0.01, 0.20; *P < .*0001; Fig. 2C).

The adjusted median TTD in QoL was NR (95% CI: 2.5 months, NR) for dostarlimab and 4.5 months (95% CI: 4.1, 5.4) for doxorubicin (HR 0.58; *P = .*0011; Fig. 2D).

### Safety

AEs during the GARNET and ZoptEC studies were reported independently and were consistent with known AEs related to dostarlimab and doxorubicin.

There were similar rates of SAEs with dostarlimab and doxorubicin, reported in 34.1% (95% CI: 26.0, 43.0) and 30.1% (95% CI: 24.5, 36.2) of patients, respectively (adjusted OR: 1.30 [95% CI: 0.77, 2.19; *P = .*321], and RR: 1.20 [95% CI: 0.84, 1.71; *P = .*314) (Table 2). The rates of TEAEs leading to treatment discontinuation for dostarlimab (11.6% [95% CI: 6.7, 18.5]) and doxorubicin (15.3% [95% CI: 11.0, 20.3) were also comparable (adjusted OR: 0.49 [95% CI: 0.21, 1.13; *P = .*094], and RR: 0.53 [95% CI: 0.24, 1.14; *P = .*101]). CTCAE Grade ≥3 TEAEs were reported by a lower proportion of patients who received dostarlimab than doxorubicin, occurring in 48.1% (95% CI: 39.2, 57.0) and 78.3% (95% CI: 72.7, 83.3) of patients, respectively (adjusted OR: 0.29 [95% CI: 0.17, 0.47; *P < .*0001], and RR: 0.63 [95% CI: 0.51, 0.79; *P < .*0001) (Table 2). The most common CTCAE Grade ≥3 TEAE (occurring in ≥5% of patients) was anemia in GARNET (14.7%) and neutropenia (45.0%) in ZoptEC (Table 3).

**Table 2.** Summary of AEs with dostarlimab and doxorubicin.

	Dostarlimab (*N* = 129)	Doxorubicin (*N* = 249)
Serious AEs		
No. of SAEs	44	75
Proportion of SAEs (95% CI)	0.341 (0.260, 0.430)	0.301 (0.245, 0.362)
Adjusted matched OR (95% CI; *P*-value)^a^	1.30 (0.77, 2.19; *P = .*321)	
Adjusted matched RR (95% CI; *P*-value)^a^	1.20 (0.84, 1.71; *P = .*314)	
TEAEs leading to treatment discontinuation		
No. of TEAEs leading to treatment discontinuation	15	38
Proportion of TEAEs (95% CI)	0.116 (0.067, 0.185)	0.153 (0.110, 0.203)
Adjusted matched OR (95% CI; *P*-value)^a^	0.49 (0.21, 1.13; *P = .*094)	
Adjusted matched RR (95% CI; *P*-value)^a^	0.53 (0.24, 1.14; *P = .*101)	
CTCAE Grade ≥3		
No. of CTCAE Grade ≥3	62	195
Proportion of CTCAE Grade ≥3 (95% CI)	0.481 (0.392, 0.570)	0.783 (0.727, 0.833)
Adjusted matched OR (95% CI; *P*-value)^a^	0.29 (0.17, 0.47; *P < .*0001)	
Adjusted matched RR (95% CI; *P-*value)^a^	0.63 (0.51, 0.79; *P < .*0001)	

Dostarlimab/doxorubicin.

Abbreviations: AE, adverse event; CI, confidence interval; CTCAE, Common Terminology Criteria for Adverse Events; OR, odds ratio; RR, relative risk; SAE, serious adverse event; TEAE, treatment-emergent adverse event.

**Table 3.** TEAEs Grade ≥3 per CTCAE occurring with a ≥5% frequency in either the GARNET or ZoptEC studies.

CTCAE grade ≥3, *n* (%)	Dostarlimab (*N* = 129)	Doxorubicin (*N* = 249)
Abdominal pain	7 (5.4)	4 (1.6)
Anemia	19 (14.7)	38 (15.3)
Fatigue	1 (0.8)	14 (5.6)
Leukopenia	2 (1.6)	45 (18.1)
Nausea	NR	13 (5.2)
Neutropenia	2 (1.6)	112 (45.0)
Neutrophil count decreased	NR	25 (10.0)
Vomiting	NR	13 (5.2)
White blood cell count decreased	NR	20 (8.0)

Abbreviations: CTCAE, Common Terminology Criteria for Adverse Events; NR, not reported; TEAE, treatment-emergent adverse event.

## Discussion

The matching procedure used by IPTW in this ITC demonstrated that treatment with dostarlimab resulted in a significantly increased OS relative to doxorubicin, with a 59% lower risk of death in patients with advanced/recurrent EC. The median OS for dostarlimab was not reached in GARNET, but the trajectory of the Kaplan-Meier curve for OS suggests that with longer follow-up the estimated HR could improve further for dostarlimab relative to doxorubicin. A greater treatment effect in terms of OS was observed for dostarlimab compared with doxorubicin after approximately 5 months (Fig. 2). This was expected, as immune checkpoint inhibitors may have a slow time to response.^30^ The primary OS estimate was robustly validated, as demonstrated by similar results produced by the sensitivity analysis.

The use of mASM to adjust for inter-study differences in tumor assessment timepoints allowed for PFS, DoR, and TTD in QoL to be compared statistically between the 2 treatments. The median PFS was approximately 2.5 times longer for dostarlimab (12.2 vs 4.9 months) and was associated with an estimated 62% lower hazard for progression or death than doxorubicin. The rate of clinical response was approximately 3 times higher (44% vs 14%) and more durable with dostarlimab than doxorubicin, with DoR not being reached for dostarlimab, compared with 8.4 months for doxorubicin.

The efficacy results for doxorubicin in ZoptEC are comparable to those observed for other single-agent chemotherapies in patients with recurrent or advanced EC with prior platinum exposure. In a Phase III trial (*n* = 496) comparing ixabepilone with standard chemotherapy (paclitaxel or doxorubicin, depending on prior therapy received), median OS was 10.9 and 12.3 months, median PFS was 3.4 and 4.0 months, and ORR was 15.2% and 15.7%, respectively (DoR was NR).^31^ In the GOG129-P Phase II single-arm trial of ixabepilone (*n* = 50), ORR was 12%, and median OS and PFS were ≥8.7 and 2.9 months, respectively.^32^ In 2 other GOG129 studies of paclitaxel (*n* = 44)^33^ and oxaliplatin (*n* = 52),^34^ ORR was 27.3% and 13.5%, with median DoR of 4.2 and 10.9+ months, respectively (OS and PFS were NR).

Patients’ QoL is an important factor when considering treatments in advanced disease, and this ITC showed that deterioration in QoL was slower with dostarlimab than doxorubicin, potentially a reflection of the increased PFS associated with dostarlimab when compared with doxorubicin.

In terms of safety, the rates of SAEs and TEAEs leading to discontinuation were similar between treatments, but a higher proportion of patients had CTCAE Grade ≥3 TEAEs with doxorubicin than with dostarlimab.

A lower proportion of patients enrolled in GARNET had tumors with serous histology compared with patients enrolled in the control arm of ZoptEC (4.3% vs 26.2%, respectively). To ensure this did not influence OS outcomes given that serous carcinoma is associated with worse prognosis than endometroid carcinomas,^35^ the HR for OS was calculated with the exclusion of all patients with serous carcinoma from both GARNET and ZoptEC populations. The HR was similar for both the full and the serous-free population (HR: 0.41; 95% CI: 0.28-0.61; *P* ≤ .0001) and main analysis population (HR: 0.46; 95% CI: 0.31-0.68; *P* = .0001), respectively, providing confidence that the difference in patient demographics was not meaningful for the efficacy analyses using the main analyses population.

In the absence of comparative clinical studies, population-adjusted ITCs allow the comparison of endpoints between studies and are recommended by the NICE Decision Support Unit.^20^ ITC analytical methods require that the studies have similar patient populations, designs, and definitions.^28^ However, there are some inherent limitations with this type of comparison. The IPTW analysis cannot be considered a replacement for a direct head-to-head comparison in a randomized controlled trial. Despite assessment schedule matching, inter-study differences in timing of the assessments may have impacted the interpretation of the results of the time-related outcomes, and there are biases associated with this method. Additionally, a lower number of patients who received dostarlimab compared with those who received doxorubicin were used in the analysis. Lastly, there could be an unknown impact of doxorubicin on MMR/MSI status.

A strength of the ITC comparison used in this study is that because patient-level data were available from both GARNET and ZoptEC, the IPTW method used is stronger than other population-adjusted ITCs such as matching-adjusted indirect comparisons, where patient-level data are usually only available for 1 study. Additionally, the present study provides inferential value in planning future formal studies, and performance of the doxorubicin control group was similar to other chemotherapies commonly used in advanced/recurrent EC as supported by randomized trials such as KEYNOTE-775.^22^

## Conclusion

This ITC demonstrated large improvements in survival and better clinical responses with dostarlimab as compared with doxorubicin, indicating that dostarlimab may show an improved benefit-to-risk profile over doxorubicin for the treatment of dMMR/MSI-H advanced/recurrent EC. These findings may be useful to physicians and health technology assessment and reimbursement bodies when assessing the overall value of dostarlimab for the treatment of this population of patients with EC.

## Supplementary Material

oyac188_suppl_Supplementary_Tables

## Data Availability

GSK makes available anonymized individual participant data and associated documents from interventional clinical studies that evaluate medicines, upon approval of proposals submitted to www.clinicalstudydatarequest.com. To access data for other types of GSK sponsored research, for study documents without patient-level data, and for clinical studies not listed, please submit an enquiry via this website.
