# Correction to: An Indirect Comparison of the Efficacy and Safety of Dostarlimab and Doxorubicin for the Treatment of Advanced and Recurrent Endometrial Cancer

**DOI:** 10.1093/oncolo/oyac250

**Published:** 2022-11-18

This is a correction to: Cara Mathews, Domenica Lorusso, Robert L Coleman, Susan Boklage, Jamie Garside, An Indirect Comparison of the Efficacy and Safety of Dostarlimab and Doxorubicin for the Treatment of Advanced and Recurrent Endometrial Cancer, *The Oncologist*, Volume 27, Issue 12, December 2022, Pages 1058–1066, https://doi.org/10.1093/oncolo/oyac188

In the originally published version of this article, there were errors in the panels A and B of Figure 2: Dostarlimab should show a blue line and Doxorubicin should show a red line.

This error has been corrected.

