# Supplementary material for: An Indirect Comparison of the Efficacy and Safety of Dostarlimab and Doxorubicin for the Treatment of Advanced and Recurrent Endometrial Cancer
Source: Oncologist. 2022 Sep 16;27(12):1058–66. doi: 10.1093/oncolo/oyac188 (PMC9732237; doi:10.1093/oncolo/oyac188)
Supplement: oyac188_suppl_Supplementary_Tables [file oyac188_suppl_supplementary_tables.docx]

# Supplementary materials

**Table S1. Key clinical criteria for the GARNET and ZoptEC studies**

|  | **GARNET (dostarlimab)**  **(N=129)** | **ZoptEC (doxorubicin)**  **(N=249)** |
| --- | --- | --- |
| Study design | Multicenter, open-label, first-in-human, Phase I trial | Multicenter, randomized, active-controlled, Phase III trial |
| Key inclusion criteria |  | |
| Age | ≥18 | ≥18 |
| Prior anti-cancer regimens | Up to 2 prior lines | 1 prior line |
| Biomarker status | dMMR/MSI-H | Not tested |
| ECOG performance status | ≤1 | ≤2 |
| Key exclusion criteria | Patient has received prior therapy with an anti-PD-1, anti-PD-1-ligand-1 (anti-PD-L1), or  anti-PD-1 ligand-2 (anti-PD-L2) agent. | ECOG performance status >2 or inadequate hematologic, cardiac, hepatic or renal function |
| Response criteria used | RECIST v1.1 | RECIST v1.1 |

dMMR, mismatch repair deficiency; ECOG, Eastern Cooperative Oncology Group; MSI-H, microsatellite instability–high; PD-(L)1, programmed death (ligand) 1; RECIST, Response Evaluation Criteria in Solid Tumors.

**Table S2. HR for OS excluding all patients with serous histology**

| **Analysis population, n (GARNET/ZoptEC)** | **HR (95% CI)** | ***P*-value** |
| --- | --- | --- |
| 88/172 | 0.46 (0.31, 0.89) | 0.0001 |

CI, confidence interval; HR, hazard ratio; OS, overall survival.
